# Feshbach Resonances in *p*-Wave Three-Body Recombination within Fermi-Fermi Mixtures of Open-Shell ^6^Li and Closed-Shell ^173^Yb Atoms

**DOI:** 10.1103/PhysRevX.10.031037

**Published:** 2020

**Authors:** Alaina Green, Hui Li, Jun Hui See Toh, Xinxin Tang, Katherine C. McCormick, Ming Li, Eite Tiesinga, Svetlana Kotochigova, Subhadeep Gupta

**Affiliations:** 1 Department of Physics, University of Washington, Seattle, Washington 98195, USA; 2 Department of Physics, Temple University, Philadelphia, Pennsylvania 19122, USA; 3 Joint Quantum Institute and Joint Center for Quantum Information and Computer Science, National Institute of Standards and Technology and University of Maryland, Gaithersburg, Maryland 20899, USA

**Keywords:** Atomic and Molecular Physics

## Abstract

We report on the observation of magnetic Feshbach resonances in a Fermi-Fermi mixture of ultracold atoms with extreme mass imbalance and on their unique *p*-wave dominated three-body recombination processes. Our system consists of open-shell alkali-metal ^6^Li and closed-shell ^173^Yb atoms, both spin polarized and held at various temperatures between 1 and 20 *μ*K. We confirm that Feshbach resonances in this system are solely the result of a weak separation-dependent hyperfine coupling between the electronic spin of ^6^Li and the nuclear spin of ^173^Yb. Our analysis also shows that three-body recombination rates are controlled by the identical fermion nature of the mixture, even in the presence of *s*-wave collisions between the two species and with recombination rate coefficients outside the Wigner threshold regime at our lowest temperature. Specifically, a comparison of experimental and theoretical line shapes of the recombination process indicates that the characteristic asymmetric line shape as a function of applied magnetic field and a maximum recombination rate coefficient that is independent of temperature can only be explained by triatomic collisions with nonzero, *p*-wave total orbital angular momentum. The resonances can be used to form ultracold doublet ground-state molecules and to simulate quantum superfluidity in mass-imbalanced mixtures.

## I. INTRODUCTION

Magnetic Feshbach resonances (MFRs) are valuable tools in ultracold bosonic and fermionic atomic gases, providing access to tunable interactions between atoms [1,2]. First observed two decades ago [3,4], they are now routinely used in few- and many-body physics. For example, they are used in the creation of ultracold molecules [5], in studies of three-body physics [6], and to elucidate collective phenomena in Bose-Einstein condensates [7] and fermionic super-fluids [8]. Three-body recombination near MFRs leads to resonantly enhanced atom loss as well as the formation of triatomic Efimov states [9,10]. Recombination in atomic gases has been studied around weak Feshbach resonances [11,12]. For bosons, their collision-energy dependence has been examined in Ref. [13]. Three-molecule recombination has also been studied in gases of ultracold polar molecules [14].

In our research collaboration we investigate ultracold mixtures of alkali lithium and closed-shell ytterbium atoms [15-19] with their extreme mass ratio or imbalance of approximately 30. Quantum-degenerate mass-imbalanced mixtures [20-22] are of interest for several reasons. For instance, they can be used to investigate impurity physics [23-25], to study Fermi-Fermi mixtures [26-28], and to provide a platform for the simulation of the Kondo effect [29].

Here we report on the first observation of interspecies magnetic Feshbach resonances in the fermionic ^173^Yb-^6^Li mixture. The observations confirm the accuracy of the single interatomic potential controlling their interactions that we published in Ref. [19]. In addition, we show that the resonances are due to weak atom-separation-dependent hyperfine interactions and determine their strengths. Together with the use of pure spin states, i.e., spin-polarized atomic gases in the present work, the corresponding comparison between experiment and theory fully reveals the underlying coupling mechanism for the strongest resonances between alkali-metal and closed-shell atoms for the first time.

We also report on the distinctive temperature dependence of the resonant three-body recombination processes that enabled us to detect the Feshbach resonances through enhanced atom loss. We show that three-body recombination rates are controlled by the identical fermion nature of the mixture, even though *s*-wave interspecies collisions are present and even when the recombination rate coefficients are outside the Wigner threshold regime at our temperatures. In our fermionic system, recombination is dominated by trimer *p*-wave collisions and, as we show, has an asymmetric line shape and a temperature-independent maximum loss rate coefficient. Previously, such temperature-dependent studies have only been performed in bosonic systems [30-34] where the rate coefficients are controlled by either *s*- or *d*-wave trimer collisions and *p*-wave collisions do not play a role.

Feshbach resonances have been observed or predicted for several mixtures of alkali-metal and alkaline-earth atomic gases. Reference [35] describes a study of MFRs in ultracold unpolarized mixtures of bosonic ^87^Rb and ^87,88^Sr with nearly equal masses. Resonance positions for bosonic ^133^Cs and Yb systems have been predicted [36]. As shown in Refs. [37,38], the existence of the strongest of the resonances follows from weak atom-separation-dependent hyperfine interactions between electronic spin of the ^2^*S* atom and the nuclear spin of the ^1^*S* atom. Although the origin of the Feshbach resonances in our study is similar to that in ^87^Sr-^87^Rb, the observation of these resonances in our Fermi-Fermi mixture represents a significant broadening of the scope of research with fermionic systems.

The research scope is further broadened by the large mass imbalance factor of approximately 30. Interspecies Feshbach resonances in Li and Yb mixtures then form an ideal tool to study impurity physics [23-25]. For example, in a Fermi liquid with impurities near a Feshbach resonance, polarons are an important elementary excitation whose properties will determine the stability of the liquid. Such resonances can also be used to explore the *fermionic* equivalent of Efimov states [39]. Research on exotic many-body phases such as interior gap [26], Fulde-Ferrell-Larkin-Ovchinnikov [27], and breached pair [28] super-fluids will also become possible.

Finally, Feshbach resonances among alkali-metal and closed-shell atoms open up the possibility of creating ultracold heteronuclear ^2^Σ^+^ molecules [19,35,36,40-42]. With the additional degree of freedom from the unpaired electron, such molecules extend the scientific relevance of ultracold molecules, currently limited to bialkali molecules [5,43-47], with unique roles in the simulation of many-body systems, studies of quantum magnetism, fundamental symmetry tests, and ultracold chemistry [48-51].

The remainder of this paper is organized as follows. In Secs. II-VI we report on the observation and quantitative model of Feshbach resonances in spin-polarized ultracold mixtures of fermionic ^6^Li and fermionic ^173^Yb. In Secs. VII and VIII we describe the distinctive role of fermionic statistics on the thermal dependence of resonant three-body recombination near these ^173^Yb-^6^Li resonances. We derive differential equations for atom loss as well as an asymmetric line shape model for three-body recombination. The comparison with measured data obtained at various temperatures between 1 and 20 *μ*K shows that trimer *p*-wave collisions play a crucial role. We conclude and give an outlook in Sec. IX.

## II. MAGNETIC FESHBACH RESONANCES BETWEEN ^6^Li AND ^173^Yb

Feshbach resonances between ground-state ^6^Li and ^173^Yb in a magnetic field $\vec{B}$ are due to the coupling between an electronic Born-Oppenheimer (BO) potential and weak interatomic-separation-dependent hyperfine couplings [36-38]. The Hamiltonian for this system is *H* = *H*_0_ + *U*_*s–i*_(*R*), where

(1) $$H_{0}=-\frac{\hbar^{2}}{2\mu }\nabla^{2}+V(R)+H_{\text{Li}}+H_{\text{Yb}},$$

and *U*_*s–i*_(*R*) describes the weak *R*-dependent hyperfine couplings. Here, *R* is the interatomic separation, *μ* is the reduced atomic mass, *ℏ* = *h*/(2*π*), and *h* is the Planck constant. The term *V*(*R*) represents the ground-state *X*^2^Σ^+^ BO potential. The last two terms of Eq. (1) describe the individual atomic hyperfine and Zeeman Hamiltonians,

(2) $$H_{\text{Li}}=a_{\text{Li}}{\vec{s}}_{\text{Li}}\cdot {\vec{ı}}_{\text{Li}}+(g_{\text{Li}}^{e}{\vec{s}}_{\text{Li}}+g_{\text{Li}}^{\text{nuc}}{\vec{ı}}_{\text{Li}})\mu_{B}\cdot \vec{B},$$

(3) $$H_{\text{Yb}}=g_{\text{Yb}}^{\text{nuc}}\mu_{B}{\vec{ı}}_{\text{Yb}}\cdot \vec{B},$$

where the ^6^Li total electron spin is *s*_Li_ = 1/2 and its nuclear spin is *i*_Li_ = 1. The closed-shell ^173^Yb has a nuclear spin *i*_Yb_ = 5/2. The ^6^Li hyperfine coupling constant *a*_Li_ has units of energy and $g_{\text{Li}}^{e}$, $g_{\text{Li}}^{\text{nuc}}$, and $g_{\text{Yb}}^{\text{nuc}}$ are the dimensionless electronic and nuclear *g* factors of ^6^Li and ^173^Yb, respectively. Their values are found in Refs. [52,53]. Finally, *μ_B_* is the Bohr magneton.

**FIG. 1.**
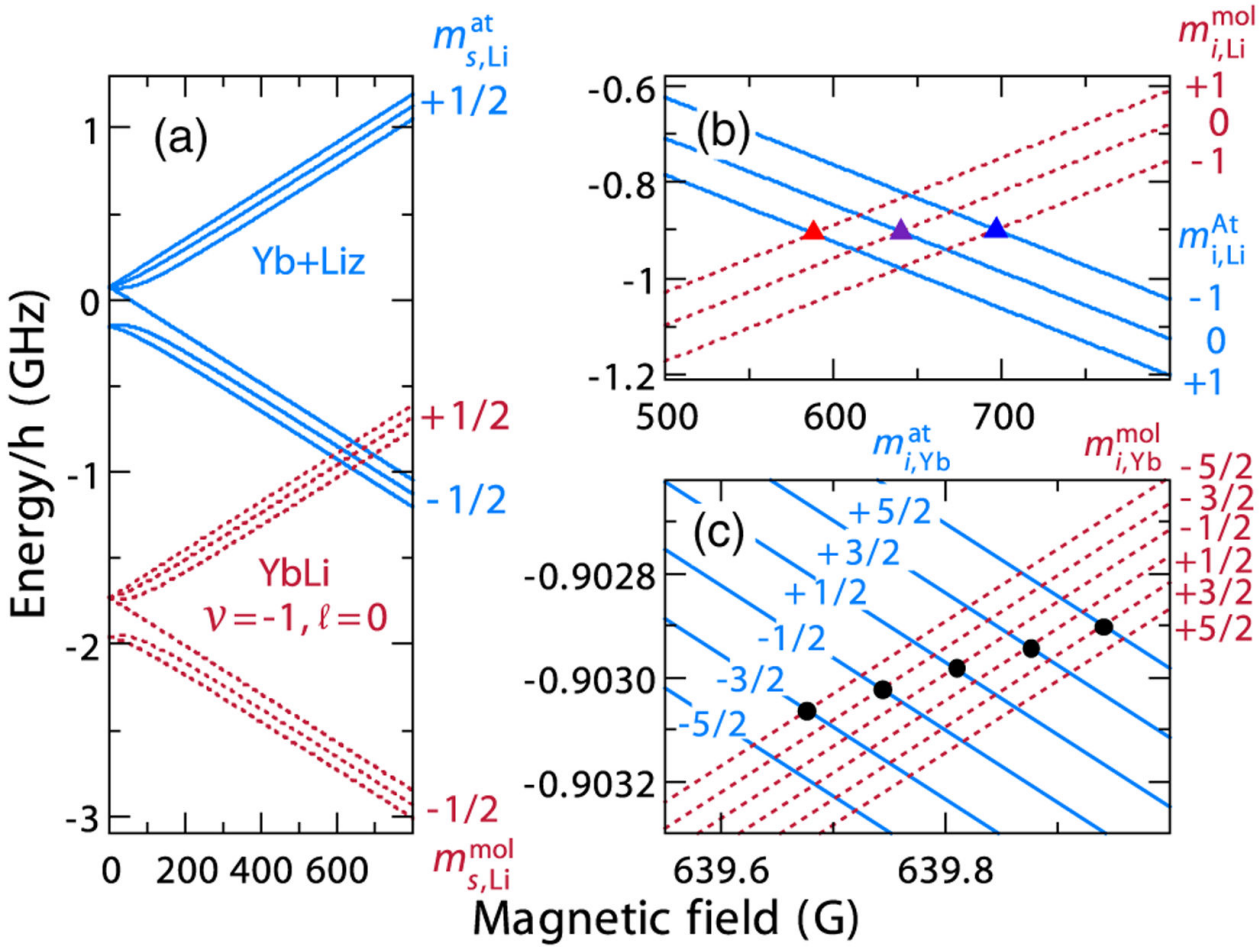
Three views of the hyperfine and Zeeman energies of ^173^Yb-^6^Li zero-energy scattering and bound states as functions of magnetic field in units of G, where 1 G is 0.1 mT. Panel (a) shows the atomic energies, eigenvalues of *H*_Li_ + *H*_Yb_, by solid blue lines and those of the most weakly bound *s*-wave bound states, eigenvalues of *H*_0_, by dashed red lines. Panel (b) shows an enlargement of the level crossing region near 640 G in (a). Panel (c) is a further enlargement of the crossing region but now shows bound-state energies of *H*_0_ + *U*_*s*–*i*_(*R*) with red dashed lines. Symbols in (b) and (c) mark the level crossings for the MFRs observed in this work. Differences in bound-state energies of Hamiltonians *H*_0_ and *H*_0_ + *U*_*s*–*i*_(*R*) would only be visible on the scales used in (c).

The ^6^Li atomic Hamiltonian *H*_Li_ has magnetic field-dependent eigenstates ∣*m*_*s*,Li_, *m*_*i*,Li_; *B*⟩ labeled by the projection of electron and nuclear spin quantum numbers along $\vec{B}$. We call this the “high-field” basis, where the *B* field label will often be suppressed in states and kets for clarity. Eigenstates of *H*_Yb_ are ∣*m*_*i*,Yb_⟩ labeled by the projection of the ^173^Yb nuclear spin quantum number along the magnetic field $\vec{B}$. The eigenvalues of *H*_Li_ + *H*_Yb_ as a function of *B* are shown in Fig. 1. The ^173^Yb nuclear Zeeman splittings are only resolved in Fig. 1(c).

The eigenstates of the *H*_0_ in Eq. (1) can be written as

(4) $$|\Psi \rangle =\phi (R)|m_{s,\text{Li}},m_{i,\text{Li}};B\rangle |m_{i,\text{Yb}}\rangle Y_{\ell m_{\ell }}(\hat{R}),$$

where spherical harmonics $Y_{\ell m_{\ell }}(\hat{R})$ describe the rotation of the two atoms with relative orbital angular momentum *ℓ* and its projection *m_ℓ_*. The radial wave function *ϕ*(*R*) is either a scattering solution of the BO potential at collision energy *E* > 0 and partial wave *ℓ* or a bound state with energy *E*_*ν, ℓ*_ < 0, where *ν* and *ℓ* are the vibrational and rotational quantum numbers, respectively. Throughout this paper these two types of solutions and spin states are distinguished by superscripts “at” and “mol” where necessary. We use the *X*^2^Σ^+^ BO potential from Ref. [19], which was obtained by fitting to six experimentally determined weakly bound states of the isotopologue ^174^Yb^6^Li. The most weakly bound state of ^173^Yb^6^Li in this potential has energy *E*_−1,0_/*h* = −1.8058(34) GHz, where the number in parentheses is the one-standard deviation uncertainty.

Bound-state energies of *H*_0_ are the sum of the ^6^Li and ^173^Yb hyperfine and Zeeman energies and *E*_*ν, ℓ*_. Binding energies of the molecular hyperfine levels with *ν* = −1 and *ℓ* = 0 as functions of *B* are shown in Figs. 1(a) and 1(b). Crossings between atomic and molecular states are visible, although the states are not coupled within *H*_0_. Once we include the weak *U*_*s*–*i*_(*R*) interactions they change into Feshbach resonances. We postpone the description of this mixing until Sec. V and first describe our experimental procedures to precisely locate the resonances.

## III. EXPERIMENTAL SETUP

We observe interspecies magnetic Feshbach resonances through enhanced atom loss over narrow ranges of magnetic field. Specifically, we measure the remaining fraction of atoms after an optically trapped spin-polarized mixture is held for a fixed time at constant magnetic field. This atom-loss spectroscopy begins with ultracold atomic samples in a crossed optical dipole trap (ODT) described in earlier work [54,55]. Unpolarized laser-cooled samples of atomic ^173^Yb are loaded into the ODT first at a bias magnetic field of ≲1 G. Subsequently, ^6^Li atoms are laser cooled, loaded into the ODT, and optically pumped into the two energetically lowest hyperfine states by applying light resonant on the ^2^*S*_1/2_ → ^2^*P*_3/2_ transition. We then increase the bias magnetic field to ≃500 G in order to spectro-scopically resolve the two remaining hyperfine states and subsequently remove atoms in the ∣*m*_*s*,Li_, *m*_*i*,Li_⟩ = ∣ −1/2, +1⟩ state with an additional resonant light pulse.

To prepare a particular spin-polarized heteronuclear mixture we use the following strategy (additional details can be found in the Supplemental Material [56]). A first stage of evaporative cooling to 5.8 *μ*K is performed by ODT depth reduction before the ^173^Yb sample is partially polarized through optical pumping into a spin mixture containing a majority of ^173^Yb atoms in state ∣*m*_*i*,Yb_ = *m*⟩ and a minority in a sacrificial state ∣*m*_*i*,Yb_ = +5/2⟩ or ∣*m*_*i*,Yb_ = −5/2⟩. The sacrificial state is retained to increase the efficiency of further evaporative cooling. Subsequently, the sample temperature is either increased or decreased to the desired value by either increasing or decreasing the ODT depth [57]. The ^173^Yb atoms in the sacrificial state are then removed with a resonant light pulse, resulting in a fully polarized ^173^Yb sample in state ∣*m*_*i*,Yb_ = *m*⟩. Finally, the atoms in the ^6^Li sample are transferred to the hyperfine ground state of interest through radio frequency (rf) adiabatic rapid passage. We have verified from a separate diagnostic that we achieve > 90% spin polarization for each atomic species in the targeted spin state [56].

Once the desired spin-polarized heteronuclear mixture is prepared, we use an approximately linear ramp to reach the desired magnetic field in less than 25 ms. We then perform loss spectroscopy at this magnetic field by letting the atoms interact for a fixed hold time, typically on the order of several seconds, after which we measure the remaining atom number through absorption imaging at 500 G. We then repeat the process for many magnetic field values. The magnetic field is generated by a pair of coils in Helmholtz configuration connected to a programmable power supply, and is calibrated through rf spectroscopy on the ^6^Li atomic ground-state hyperfine transitions. The magnetic field stability is approximately 50 mG and we verified that the loss signal is independent of the field ramp direction.

The temperature range explored in this work is between 1 and 20 μK. The differential gravitational potential in this highly mass-imbalanced system results in a partial separation of the two species at the lowest temperatures, causing a lengthening of interspecies thermalization time below ≃2 *μ*K. In this work, the lowest ^173^Yb (^6^Li) temperature at the beginning of the loss-spectroscopy phase is 1.0 *μ*K (1.8 *μ*K). This corresponds to *T/T_F_* = 3.3 (0.57), where *T_F_* is the Fermi temperature for each species. Under these conditions, the measured ^173^Yb (^6^Li) atom number at the beginning of the spectroscopy phase is 1.0 × 10^5^ (1.3 × 10^5^) with corresponding peak density of 2.6 × 10^12^ cm^−3^ (6.1 × 10^12^ cm^−3^). Here and elsewhere in this paper, the uncertainties in temperature, atom number, and density are 10%, 10%, and 18%, respectively, mainly stemming from uncertainties in the imaging system. For higher temperatures, the two species are in thermal equilibrium with each other at the beginning of the loss spectroscopy phase.

## IV. OBSERVATION OF MAGNETIC FESHBACH RESONANCES

**FIG. 2.**
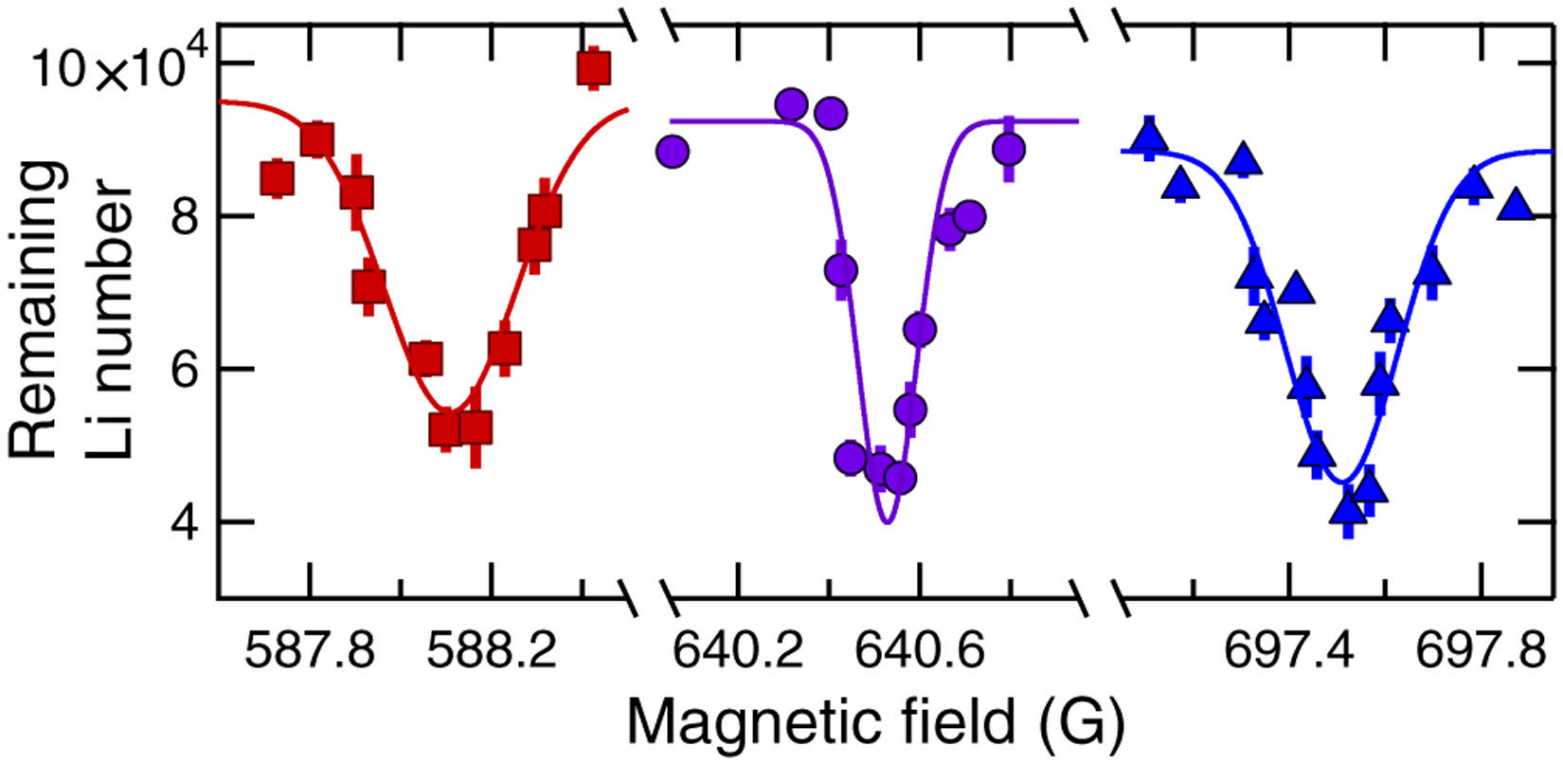
Observation of interspecies ^173^Yb^6^Li magnetic Feshbach resonances as functions of magnetic field *B*. Resonances appear as *B*-dependent atom loss. The ^6^Li atom loss is shown after the 2.8 *μ*K mixture has been held for 3 s in the dipole trap. Red squares, purple circles, and blue triangles correspond to data for ^6^Li in states ∣*m*_*s*,Li_, *m*_*i*,Li_⟩ = ∣−1/2, 1⟩, ∣−1/2, 0⟩, and ∣−1/2, −1⟩, respectively. The nuclear Zeeman state of ^173^Yb is spin polarized to ∣*m*_*i*,Yb_⟩ = ∣+5/2⟩ in each case. Each point is the average of at least four measurements and the error bars are one-standard-deviation statistical uncertainties. Curves are best-fit Gaussians and only meant as a guide to the eye. Our full line shape analysis is found in Sec. VII.

For the magnetic field range investigated in this work, three ^6^Li ground hyperfine states exhibit interspecies Feshbach resonances with ^173^Yb. These are ∣*m*_*s*,Li_, *m*_*i*,Li_⟩ = ∣−1/2, +1⟩, ∣−1/2, 0⟩, and ∣−1/2, −1⟩. Figure 2 shows the experimental observation of their interspecies Feshbach resonances when the ^173^Yb is prepared in ∣*m*_*i*,Yb_⟩ = ∣+5/2⟩. We have confirmed that the three loss features in Fig. 2 correspond to interspecies Feshbach resonances by repeating the spectroscopy phase with only ^6^Li atoms. No atom loss features were then observed.

**FIG. 3.**
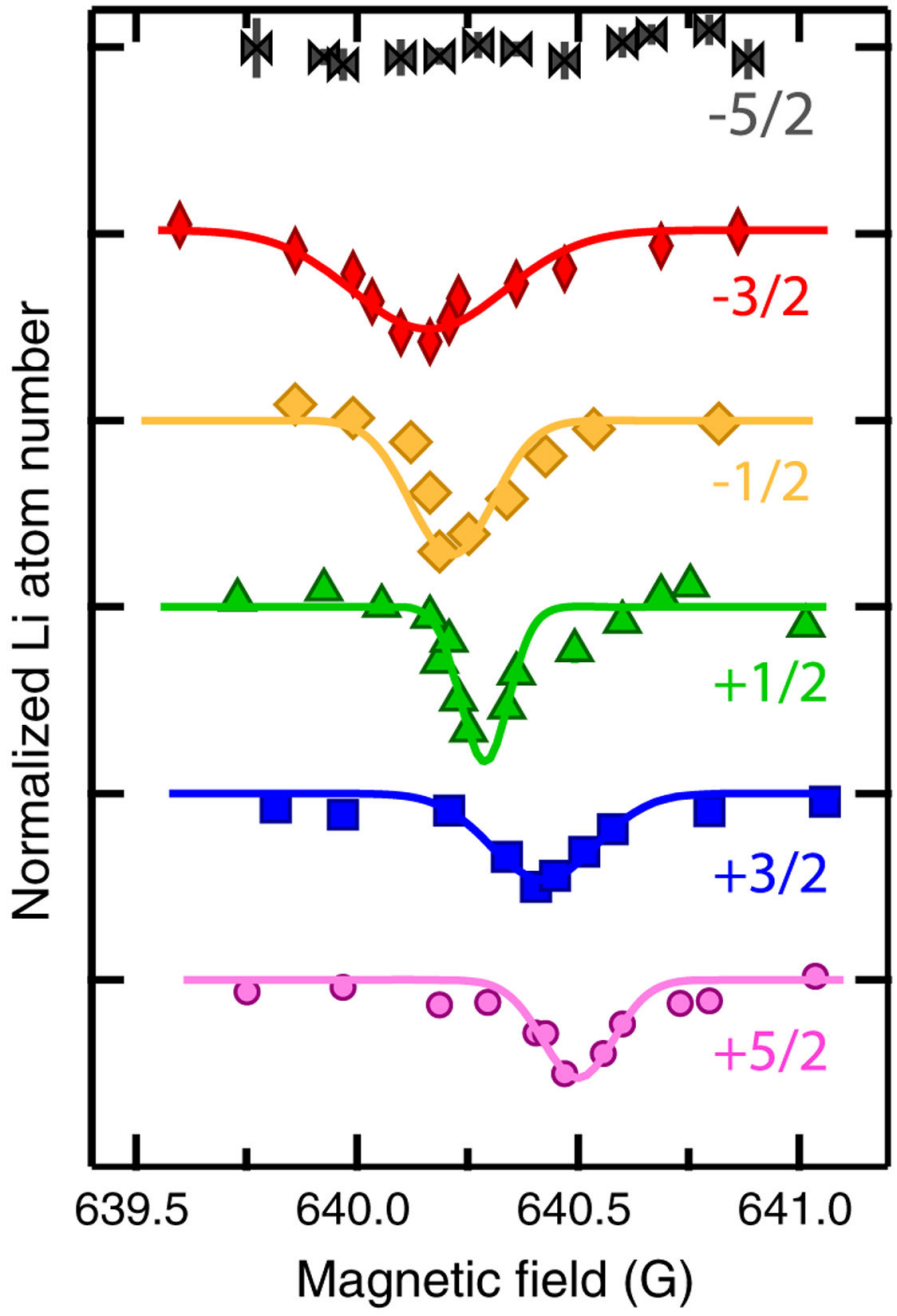
Dependence of magnetic Feshbach resonances on the ^173^Yb nuclear Zeeman state in a ^173^Yb-^6^Li mixture seen in the remaining ^6^Li atom number as functions of *B* near the 640 G Feshbach resonance. ^6^Li is prepared in hyperfine state ∣*m*_s,Li_, *m*_*i*,Li_⟩ = ∣−1/2, 0⟩ and the ^173^Yb sample is spin polarized in different nuclear Zeeman states. The data are normalized by the remaining ^6^Li atom number away from resonance and vertically offset for clarity. The distance between vertical tick marks is one. From top to bottom ^173^Yb is prepared in nuclear spin state *m*_*i*,Yb_ = −5/2, −3/2, −1/2, +1/2, +3/2, and +5/2. No resonance exists for *m*_*i*,Yb_ = −5/2. The temperature is 1.8 *μ*K (1.0 *μ*K) for Li (Yb) and the hold time is 1.5 s. Curves are best-fit Gaussians. Our full line shape analysis is found in Sec. VII.

To investigate the effect of the ^173^Yb nuclear spin on the MFRs, we repeated our trap-loss spectroscopy for each of the six *m*_*i*,Yb_ states, preparing the ^6^Li sample in ∣*m*_*s*,Li_, *m*_*i*,Li_) = ∣−1/2, 0⟩ for all cases. The results are shown in Fig. 3. The absence of a MFR for *m*_*i*,Yb_ = −5/2 is expected for reasons outlined in Sec. V.

The experimental value of the resonance location for each MFR is determined as the center value of a Gaussian fit to our lowest temperature data [58]. The locations of all observed resonances with spin-polarized heteronuclear mixtures are listed in Table I and are consistent with the predictions for Feshbach resonance locations due to the least bound state, i.e., *ν* = −1, *ℓ* = 0, of the ^2^Σ^+^ BO potential shown in Fig. 1. We present a detailed comparison of our theoretical analysis and experimental observations in Sec. VI.

**FIG. 4.**
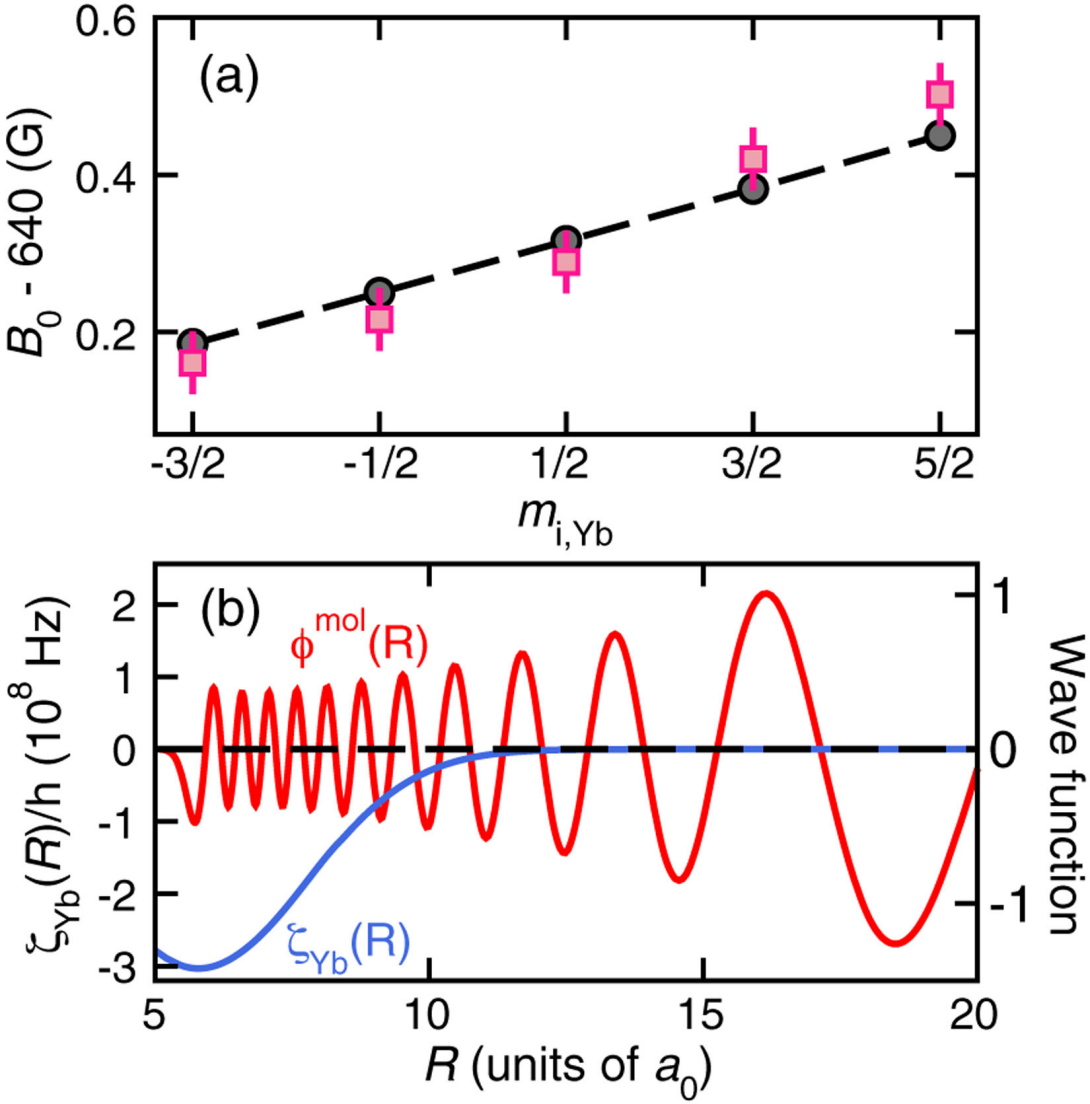
(a) The observed (pink squares) and theoretically predicted (black circles) resonance locations near *B* = 640 G as functions of *m*_*i*,Yb_. The ^6^Li atom is in state ∣*m*_*s*,Li_, *m*_*i*,Li_⟩ = ∣−1/2, 0⟩. For better visual comparison, the theoretical values have been uniformly shifted from those shown in Fig. 1(c). The shift is consistent with the 1.3 G uncertainty in the predicted resonance location. The dashed line is given by Eq. (7). (b) The ^173^Yb^6^Li hyperfine coupling coefficient *ζ*_Yb_(*R*) (solid blue line) and the radial wave function *ϕ*^mol^(*R*) of the most weakly bound state of the ^2^Σ^+^ potential (solid red line) as functions of separation *R*. Here, *a*_0_ = 0.05292 nm is the Bohr radius.

The locations of the five resonances in Fig. 3 are also indicated in Fig. 4(a). From a linear fit to these data we derive the effective magnetic moment of the “dressed” ^173^Yb nucleus in the *ν* = −1, *ℓ* = 0 ^173^Yb^6^Li molecule and note that its sign is opposite that of a ^173^Yb atom. In Secs. V and VI we show and discuss in detail that the sign change originates from the *R*-dependent *U*_*s*−*i*_(*R*) hyperfine coupling.

## V. SEPARATION-DEPENDENT HYPERFINE INTERACTIONS

We now define the weak interaction *U*_*s*–*i*_(*R*) that leads to coupling between eigenstates of *H*_0_ and hence our observed Feshbach resonances. The interaction describes the effects of the modified electron spin densities at the nuclear positions of ^6^Li and ^173^Yb when the atoms are in close proximity. For our experiment the relevant coupling is isotropic and given by [37]

(5) $$U_{s-i}(R)=\zeta_{\text{Yb}}(R){\vec{s}}_{\text{Li}}\cdot {\vec{ı}}_{\text{Yb}},$$

where the hyperfine coupling coefficient *ζ*_Yb_(*R*) is obtained from an all-electron *ab initio* calculation based on the non-relativistic configuration interaction valence-bond (CIVB) method [59-61]. Figure 4(b) shows *ζ*_Yb_(*R*) together with the real-valued radial wave function of the most weakly bound state of the ^2^Σ^+^ BO potential as a function of interatomic separation. The *ζ*_Yb_(*R*) is on the order of *a*_Li_ near the inner point, *R* ≈ 6*a*_0_, of the vibrational bound-state wave function and then approaches zero rapidly when *R* → ∞. The uncertainty of *ζ*_Yb_(*R*) at *R* = 6*a*_0_ is 4% based CIVB calculations with different basis set size as well as our ability to reproduce the experimental ^6^Li hyperfine constant *a*_Li_ to about 1%.

The weak *U*_*s*–*i*_(*R*) changes the crossings between the atomic and molecular levels in Fig. 1 into resonances. For this interaction to lead to a resonance the sum *m*_*s*,Li_ + *m*_*i*,Yb_ must be the same for the scattering and bound states. In particular, since the MFRs we investigate satisfy $m_{s,\text{Li}}^{\text{at}}(m_{s,\text{Li}}^{\text{mol}})=-1∕2(+1∕2)$ for the scattering (bound) states, no MFR is expected for $m_{i,\text{Yb}}^{\text{at}}=-5∕2$. Additionally, the projection *M*_tot_ = *m*_*s*,Li_ + *m*_*i*,Li_ + *m*_*i*,Yb_ is always conserved.

The weak *U*_*s*–*i*_(*R*) also modifies the energies of the hyperfine and Zeeman states of the *ν* = −1, *ℓ* = 0 bound state. The size of these energy shifts can only be observed over magnetic field intervals of less than 1 G, as shown in Fig. 1(c) for resonances around 640 G. These resonances are labeled by the ^6^Li state $|m_{s,\text{Li}}^{\text{at}},m_{i,\text{Li}}^{\text{at}};B\rangle =|-1∕2,0;B\rangle$ and ^173^Yb Zeeman states $|m_{i,\text{Yb}}^{\text{at}}\rangle$ for the scattering states and $|m_{s,\text{Li}}^{\text{mol}},m_{i,\text{Li}}^{\text{mol}};B\rangle =|1∕2,0;B\rangle$ and $|m_{i,\text{Yb}}^{\text{mol}}\rangle$ for the molecular states.

To get an intuitive understanding of the molecular level splitting, we perturbatively study the energies of these six bound states. The states of different *m*_*i*,Yb_ are split by the nuclear Zeeman interaction of ^173^Yb,

(6) $$\Delta E_{0}=m_{i,\text{Yb}}g_{\text{Yb}}^{\text{nuc}}\mu_{B}B,$$

and a contribution from the diagonal matrix elements of Eq. (5),

(7) $$\Delta E_{1}=\langle \Psi^{\text{mol}}|\zeta_{\text{Yb}}(R){\vec{s}}_{\text{Li}}\cdot {\vec{ı}}_{\text{Yb}}|\Psi^{\text{mol}}\rangle \;\;≃\frac{1}{2}m_{i,\text{Yb}}\int_{0}^{\infty }dR\phi^{\text{mol}}(R)\zeta_{\text{Yb}}(R)\phi^{\text{mol}}(R),$$

where for the last equality we use the fact that the magnetic field is large. The radial integral over *ζ*_Yb_(*R*) is *h* × −0.369(24) MHz for our CIVB values. The theoretical uncertainty combines in quadrature the 4% uncertainty in *ζ*_Yb_(*R*) and a 5% uncertainty due to the uncertainty in the shape of the *v* = −1, *ℓ* = 0 vibrational wave function from the *h* × 3.4 MHz uncertainty of its binding energy. Both Eqs. (6) and (7) are proportional to *m*_*i*,Yb_ and the two contributions have opposite signs. For *B* = 640 G, ∣Δ*E*_1_∣ is about 30% larger than Δ*E*_0_, resulting in an overall change in the sign of the level shifts as compared to shifts of the free-atom state.

Figure 1(c) shows the theoretical energies of the atomic levels crossing with the molecular bound states including the corrections Δ*E*_0_ and Δ*E*_1_. The energies of scattering states are not affected by *U*_*s*–*i*_(*R*). Crossings with markers in Fig. 1(c) correspond to resonances satisfying the selection rules of *U*_*s*–*i*_(*R*). Without the correction of Eq. (7) in molecular state energies, all crossings would occur at the same magnetic field [36].

We use our coupled-channels code to determine the strength and resonance locations of the MFRs and binding energies of rovibrational levels. Specifically, we compute the zero-energy *s*-wave scattering length *a*(*B*) as a function of magnetic field [62] for all relevant scattering channels $|m_{s,\text{Li}}^{\text{at}},m_{i,\text{Li}}^{\text{at}}\rangle$. Near each resonance, we fit to *a*(*B*) = *a*_bg_[1 – Δ/(*B* – *B*_res_)], where *B*_res_ is the resonance location, *a*_bg_ is the background scattering length, and Δ is the resonance strength [63].

Two other, weaker *R*-dependent hyperfine coupling terms exist in ^173^Yb^6^Li [36-38]. The first of these two is also isotropic and discussed in the Supplemental Material [56]. Predictions for resonance locations and strengths due to this coupling term are also given there. We do not discuss a final and weakest coupling term. It is anisotropic and would lead to *ℓ* = 2, *d*-wave Feshbach resonances between 50 and 100 G. Resonances due to these two coupling terms could not be observed given the limits of our signal-to-noise ratio.

## VI. RESONANCE LOCATIONS IN THEORY AND EXPERIMENT

Table I lists our observed resonance locations as well as the corresponding theoretical predictions of resonance locations, strengths, and other properties based on the BO potential that gives *E*_−10_/*h* = −1.8058 GHz for the most weakly bound state of ^173^Yb^6^Li. Near 640 G, we experimentally located the resonances for all nuclear Zeeman states of ^173^Yb. No resonance exists for *m*_*i*,Yb_ = −5/2. Similar families of resonances occur near 588 and 697 G (see Supplemental Material [56]). We note that the observed and theoretical locations are consistent, as the 3.4 MHz uncertainty in the binding energy of the *ν* = −1 and *ℓ* = 0 state and the approximately *δμ* = 1.99*μ*_*B*_ magnetic moment difference between the bound and scattering states near *B* = 640 G leads to a 1.3 G uncertainty in the theoretical resonance location. All observed locations occur at a larger magnetic field value than those of the theoretical predictions, indicating that the binding energy ∣*E*_−1,0_∣ is slightly underestimated.

Following Ref. [1] the Feshbach resonances in Table I can be classified as either open or closed channel dominated (and less precisely as strong and weak) based on whether dimensionless $s_{\text{res}}=a_{\text{bg}}\delta \mu \Delta ∕(\bar{a}\;\bar{E})$ is much larger or smaller than one, respectively. Here, $\bar{a}$ and $\bar{E}=\hbar^{2}∕(2\mu {\bar{a}}^{2})$ are the mean scattering length and energy for an attractive van der Waals potential with dispersion coefficient *C*_6_. For YbLi $C_{6}=1581E_{h}a_{0}^{6}$ [19], where *E*_h_ is the Hartree energy. We find *s*_res_ = 9.9 × 10^−8^ × Δ/(1 *μ*G), indicating that these observed resonances are closed-channel dominated.

Figure 4(a) compares the experimental and theoretical resonance locations near 640 G as functions of *m*_*i*,Yb_. For better visual comparison, the theoretical values have been uniformly shifted up by 0.58 G, a value within the 1.3 G uncertainty. The theoretical locations have a linear dependence on *m*_*i*,Yb_ with a slope solely determined by Eq. (7). In fact, the experimental slope of 0.088(13) G is consistent, well within two standard deviations, with the theoretical slope of 0.0666(43) G using the theoretical estimate of the magnetic moment difference.

## VII. FERMIONIC FEATURES IN RESONANT THREE-BODY RECOMBINATION

Our atom-loss measurements also confirm the fermionic statistical properties of our mixture. The requirement of an antisymmetric scattering wave function under interchange of identical fermions leads to line shapes for the three-body loss rate coefficient that are controlled by *p*-wave scattering. Specifically, we show based on a theoretical model for three-body recombination that the *B*-field locations of strongest atom loss shift linearly with increasing temperature, which can only be explained by the fermionic nature of the scattering atoms. In addition, we show that our data are consistent with a second prediction of the model. The maximum value of the recombination rate coefficient is independent of temperature for the 1–20 *μ*K experimental temperature range, in sharp contrast to a linear dependence with temperature based on the *p*-wave Wigner threshold limit.

We start by noting that atom loss from our mixture at temperature *T* is described by the two-coupled equations,

(8a) $$\frac{dN_{\text{Li}}}{dt}=-\Gamma_{\text{Li}}N_{\text{Li}}-2\gamma_{1}N_{\text{Li}}^{2}N_{\text{Yb}}-\gamma_{2}N_{\text{Li}}N_{\text{Yb}}^{2},$$

(8b) $$\frac{dN_{\text{Yb}}}{dt}=-\Gamma_{\text{Yb}}N_{\text{Yb}}-\gamma_{1}N_{\text{Li}}^{2}N_{\text{Yb}}-2\gamma_{2}N_{\text{Li}}N_{\text{Yb}}^{2},$$

where atom numbers *N_a_* for *a* = Li or Yb are time dependent. Rates Γ_*a*_ describe one-body background-collision-induced losses, while event rates *γ*_1_ and *γ*_2_ describe the three-body recombination processes starting from ^6^Li + ^6^Li + ^173^Yb and ^6^Li + ^173^Yb + ^173^Yb collisions, respectively. For both processes two of the three atoms are identical fermions. Finally, *γ_i_* = *K_i_*(*B*, *T*)/*V_i_*, with *i* = 1 or 2, and temperature-dependent hypervolumes,

$$\frac{1}{V_{1}}=\int d^{3}x\rho_{\text{Li}}^{2}(\vec{x})\rho_{\text{Yb}}(\vec{x})\;\text{and}\;\frac{1}{V_{2}}=\int d^{3}x\rho_{\text{Li}}(\vec{x})\rho_{\text{Yb}}^{2}(\vec{x}),$$

where the time-independent $\rho_{a}(\vec{x})$ are unit-normalized spatial density profiles. The *event* rate coefficients *K_i_*(*B*, *T*) are discussed below.

Several assumptions have gone into deriving Eqs. (8) from two coupled Boltzmann equations for the single-particle phase-space densities $f_{a}(\vec{x},\vec{p},t)$ [64,65] with momentum $\vec{p}$. We assume that both the fermionic ^6^Li and ^173^Yb gases are in thermal equilibrium at a temperature above degeneracy and $f_{a}(\vec{x},\vec{p},t)\propto N_{a}(t)\rho_{a}(\vec{x})\;\text{exp}[-p^{2}∕(2m_{a}k_{B}T)]$, where *m_a_* is the mass of atom *a* and *k_B_* is the Boltzmann constant. This is justified as the mean time between thermalizing elastic Yb + Li collisions is much smaller than the timescales of atom loss due to three-body recombination [19,20,66]. Even though the two species are held in the same dipole trap, their spatial density profiles $\rho_{a}(\vec{x})$ are distinct as their dynamic polarizabilities and gravitational potentials are different. In this section, however, the small differences in temperature and spatial density profiles between the ^6^Li and ^173^Yb gases will be ignored. In fact, differences are only significant for our smallest measured temperature, where quantum degeneracy is almost reached and the thermalization times are longest. Finally, losses from two-body Li + Yb collisions are negligible, as confirmed by our coupled-channels calculations. Other two- and three-body losses are suppressed by the fermionic nature of the ^6^Li and ^173^Yb atoms.

The three-body recombination event rate coefficient *K_i_*(*B*, *T*) has a modified Lorentzian form as a function of *B* describing the temporary formation of a resonant trimer from three scattering atoms followed by breakup into a weakly bound dimer and a free atom. That is [67,68],

(9) $$K_{i}(B,T)=\langle (2J+1)\frac{\hbar k}{\mu_{3}}\frac{2^{5}\pi^{2}}{k^{5}}|S_{J}(B,E){|}^{2}\rangle ,$$

where ⟨ ⋯ ⟩ represents the three-particle thermal average, the relative kinetic energy $E=\hbar^{2}k^{2}∕(2\mu_{3}),\;\mu_{3}=\sqrt{m_{1}m_{2}m_{3}∕(m_{1}+m_{2}+m_{3})}$ is the three-body reduced mass, *k* is the relative wave number, and *m_a_* with *a* = 1, 2, 3 are the masses of the individual atoms. (For historical context, see also Refs. [69,70].) For our low temperatures, only the lowest-allowed total three-body angular momentum *J* contributes to atom loss. We have *J* = 1 or *p*-wave collisions for our Fermi-Fermi mixture. For later comparisons we note that the lowest angular momentum *J* is zero or two in spin-polarized single-species Bose gases. It is *J* = 0 for Feshbach resonances that occur in *s*-wave entrance-channel atom-atom collisions and *J* = 2 for Feshbach resonances that occur in *d*-wave entrance-channel atom-atom collisions, respectively [31].

The absolute value squared of the inelastic *S*-matrix element *S_J_*(*B*, *E*) is a Breit-Wigner or Fano line profile for ultracold scattering from an isolated resonance. Assuming that three-body recombination is negligible away from the resonance, it is given by [69,71-73]

(10) $$|S_{J}(B,E){|}^{2}=\frac{\Gamma (E,J)\Gamma_{\text{br}}}{(E-E_{0}{)}^{2}+(\Gamma (E,J)+\Gamma_{\text{br}}{)}^{2}∕4},$$

with *E*_0_ = *δμ*_3_(*B* – *B*_0_), where *B*_0_ and *δμ*_3_ are the three-body resonance location and the relative magnetic moment, respectively. *A priori*, the three-body *B*_0_ and two-body *B*_res_ resonance locations need not be the same, although we can assume that *B*_0_ and *B*_res_ agree to within the experimental uncertainty. The “stimulated” width Γ(*E*, *J*) depends on collisions energy, omitted in the usual formulation of the Fano profile, and is given by

(11) $$\Gamma (E,J)=A(E∕E_{\text{ref}}{)}^{2+J},$$

with scaled energy width *A* and a convenient reference energy *E*_ref_. The stimulated width has a power-law or Wigner threshold energy dependence reflecting the energy dependence of the underlying matrix element of three ultracold *p*-wave scattering atoms coupling to the resonant trimer state. Finally, Γ_br_ is the energy-independent breakup width of the resonant trimer falling apart into a stable diatomic molecule and a fast free atom. The kinetic energy of the dimer and free atom is much larger than the depth of our optical dipole trap and both atom and molecule are lost.

The resonant trimer state is only formed temporarily and should not be confused with Efimov states [67]. The latter are (stable) bound states of the three atoms with energy *E* < 0. Their wave functions are superpositions of the resonant trimer state and three-atom scattering wave functions. The resonant trimer state is spatially localized with a size that is much smaller than that of an Efimov state.

We now make several simplifications of Eqs. (8) consistent with our experimental system parameters. We use that the initial atom number and peak density of the two species are the same to good approximation and that the one-body loss rates satisfy Γ_Li_ ≃ Γ_Yb_ = Γ_bg_ [74]. Similarly, for the three-body rate we assume that *K*_1_(*B*, *T*) = *K*_2_(*B*, *T*) = *K*(*B*, *T*) and $1∕V_{1}=1∕V_{2}=\rho_{\text{Li}}^{2}(\vec{x}=\vec{0})$ as both processes involve fermions that have roughly the same phase-space density and $\vec{x}=\vec{0}$ is the center of the trap. Then Eqs. (8) become

(12) $$\frac{dN_{a}}{dt}=-\Gamma_{\text{bg}}N_{a}-3\gamma N_{a}^{3},$$

for both *a* = Li and Yb. This differential equation has an analytic solution.

We note that with Eq. (12) we have opted for the simplest model that still captures the relevant physics. In particular, there exists no formal justification for our choice *K*_1_(*B*, *T*) = *K*_2_(*B*, *T*). We are also unable to experimentally distinguish the two processes. In a recent experiment with a Fermi-Fermi mixture [75], the two processes could also not be distinguished. Reports on three-body recombination in Bose-Bose [76,77] and Bose-Fermi [78-80] mixtures showed that the light + heavy + heavy process is much faster than the light + light + heavy one.

## VIII. ANALYSIS OF LINE SHAPE OF THREE-BODY RECOMBINATION

**FIG. 5.**
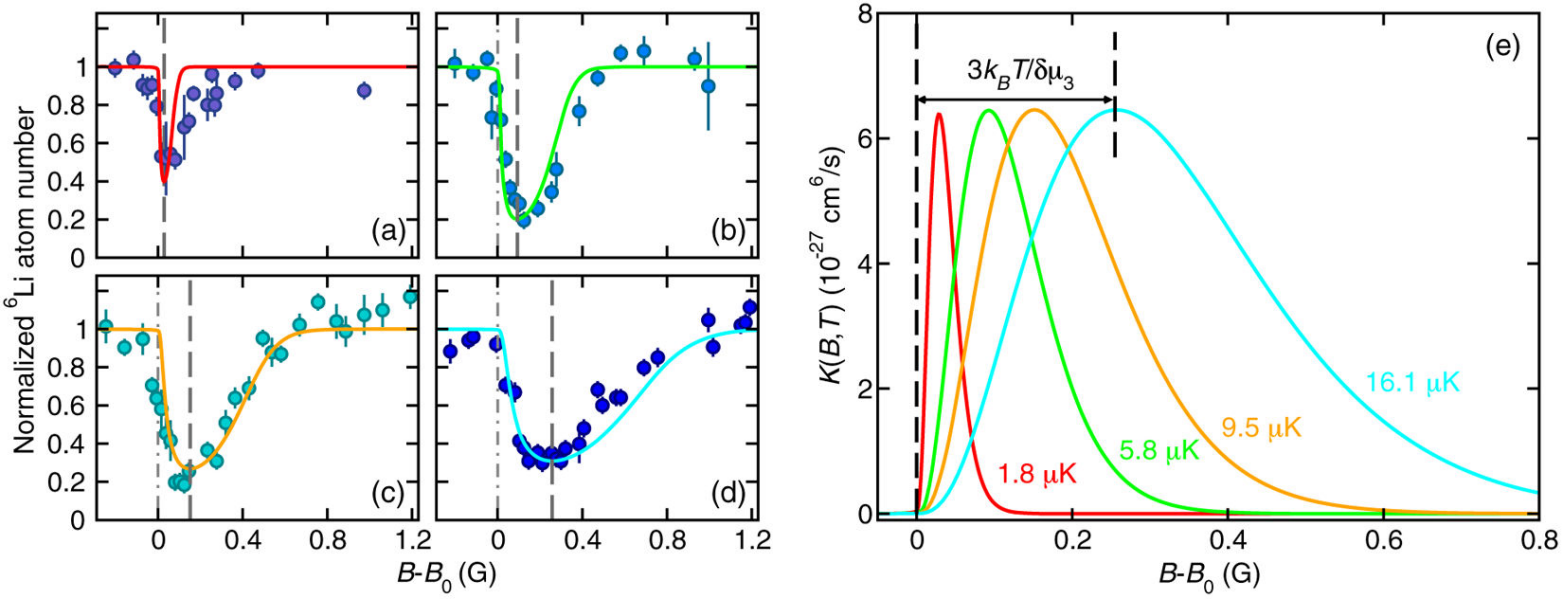
Fermionic behavior of three-body recombination processes in ^6^Li and ^173^Yb mixtures found in atom-loss spectra and fitted theoretical line shapes as functions of magnetic field. ^6^Li is prepared in the ∣*m*_*s*,Li_, *m*_*i*,Li_) = ∣− 1/2, 0⟩ state and ^173^Yb in the ∣*m*_*i*,Yb_⟩ = ∣+5/2⟩ state. The hold time is 4 s. Panels (a)–(d) correspond to the remaining ^6^Li atom number normalized to the remaining fitted ^6^Li atom number away from the resonance (markers with error bars) as a function of *B* – *B*_0_ measured at temperatures of {1.8, 5.8, 9.5, 16.1} *μ*K, respectively. The initial atom numbers for Li (Yb) are {1.3(1.0), 1.7(3.3), 1.3, (2.0), 1.8(4.0)} × 10^5^ and the initial peak densities for Li (Yb) are {6.1(2.6), 12(8.3), 10(6.2), 11(9.3)} × 10^12^ cm^−3^. The fitted theoretical line shapes are the colored curves. We specify the magnetic field relative to the resonance location *B*_0_ as determined from the fit. The dash-dotted and dashed lines, where given, locate the fitted resonance location *B*_0_ and the field of maximum atom loss, respectively. The vertical axes are scaled to the theoretical background values away from the resonance. The magnetic moment of the trimer resonance state is *δμ*_3_ = 2.8*μ_B_*, while *B*_0_ = 640.476 G, *A/k_B_* = 6 × 10^−6^ nK, and Γ_br_/*k_B_* = 100 nK. The one-body loss rate is Γ_bg_ = 0.1 s^−1^ for our lowest temperature and 0.075 s^−1^ otherwise. Panel (e) shows the theoretical event rate coefficients as functions of *B* – *B*_0_ for our four temperatures. The line colors correspond to those used in (a)–(d).

Figures 5(a) - 5(d) show atom-loss spectra, i.e., remaining atom number *N_a_* (*t_h_*) versus *B* after hold time *t_h_*, and fitted theoretical line shapes based on Eq. (12) and the three-body event rate coefficient derived from Eqs. (9) and (10) for a mixture with ^6^Li prepared in ∣*m*_*s*,Li_, *m*_*i*,Li_⟩ = ∣−1/2, 0⟩ and ^173^Yb prepared in ∣*m*_*i*,Yb_⟩ = ∣+5/2⟩. Data are shown for four temperatures between 1.8 and 16.1 *μ*K. Figure 5(e) compares the corresponding theoretical event rate coefficients *K*(*B*, *T*) as functions of *B*. While the three-body recombination process also causes heating of the atomic clouds, the measured temperature increase remains within 20% during the first second of hold time when the three-body-induced atom loss dominates over other losses.

The temperature-independent parameters *Γ*_bg_, *B*_0_, *δμ*_3_, *A*, and *Γ*_br_ in Eq. (10) are determined by simultaneous fitting of the four observed line shapes, except for the data taken at 1.8 *μ*K, where the one-body loss rate is 25% faster. For this temperature some of our theoretical assumptions also begin to break down, as noted earlier. We have used *E*_ref_/*k*_B_ = 1 *μ*K for all fits. This simply reflects the temperature range of our atomic gases.

The satisfactory agreement between experimental data and theoretical line shapes in Fig. 5 enables us to extract the *p*-wave character of the event rate coefficients. We first observe that the atom-loss features are asymmetric, that the location of maximum atom loss shifts to larger *B* with increasing temperature, and that the lines broaden with increasing temperature. Then from Fig. 5(e) we also note that the maximum event rate coefficient is independent of temperature for our four temperatures.

These behaviors directly follow from Eq. (9) and the relationships between the fitted parameters. In fact, for our parameters, given in the caption of Fig. 5, the inequalities Γ(*E*, *J*) ≪ Γ_br_ ≪ *k_B_T* hold for collision energies *E* up to several *k_B_T*. We call this the thermally limited regime, which must be distinguished from the situation where *k_B_T* ≪ Γ_br_. The latter limit corresponds to temperatures below 100 nK for our mixture. At such low temperatures our Fermi-Fermi mixture will be quantum degenerate and the model assumptions are invalid.

For both limiting cases approximations can be used to obtain a more intuitive understanding of the line shapes. The approximations are related to those used to analyze photo-association line shapes of two ultracold atoms [73,81]. In fact, in the thermally limited regime ∣*S_J_*(*B*, *E*)∣^2^ can be replaced by a Dirac delta function in *E* – *E*_0_ so that for *B* > *B*_0_ the rate coefficient for *J* = 1 simplifies to

(13) $$K(B,T)\propto \epsilon^{3}e^{-\epsilon },$$

with dimensionless *ϵ* = *δμ*_3_(*B* – *B*_0_)/*k_B_T* and captures all temperature and *B*-field dependence. By inspection we see that *K*(*B*, *T*) is maximal at *B* = *B*_max_ ≡ *B*_0_ + 3*k_B_T*/*δμ*_3_ with a value that is independent of temperature, confirming the results shown in Fig. 5(e). The width of the resonance features scale as *k_B_T/δμ*_3_.

Even though our model is invalid for *k_B_T* ≪ Γ_br_, it is still worth noting that the model predicts that *K*(*B*, *T*) for *J* = 1 reduces to a Lorentzian with a width given by Γ_br_ and a maximum loss rate that is proportional to *k_B_T*.

Finally, we find that the magnetic moment of the trimer resonance is *δμ*_3_ = 2.8*μ*_*B*_, which can be compared to the *δμ* = 1.99*μ_B_* magnetic moment of the diatomic ^173^Yb^6^Li resonance. We expect that the trimer magnetic moment *δμ*_3_ lies between *δμ* and 2*δμ*, corresponding to a superposition state of only one ^173^Yb^6^Li pair in the dimer resonant state and two ^173^Yb^6^Li pairs in the resonant state.

For our lowest experimental temperature, the residual shift *B*_max_ – *B*_0_ is still significant compared to our approximately 40 mG uncertainty in locating the magnetic field with maximum atom loss, although we must recall that the three-body *B*_0_ and two-body *B*_res_ do not need to occur at the same value. These observations set limits on any attempt to improve the shape of the *X*^2^Σ^+^ potential.

We can contrast our event rate coefficients in a Fermi-Fermi mixture with a similar analysis of Bose gases and Bose-Bose mixtures. References [30,31] showed that the maximum loss rate for bosonic three-body recombination near *s*- and *d*-wave Feshbach resonances scales as 1/(*k_B_T*) and *k_B_T*, respectively, in the thermally limited regime rather than the constant value observed here. This follows from the observation that the total trimer angular momentum is *J* = 0 and 2, respectively. Finally, Fig. 5(e) shows that the maximum three-body loss rate coefficient is relatively small for resonant processes, on the order of a few times 10^−27^ cm^6^/s, and similar to recently reported values for the fermionic ^40^K and ^162^Dy mixture [75].

## IX. CONCLUSION AND OUTLOOK

We have experimentally and theoretically studied the resonant scattering of ultracold fermionic ^6^Li and ^173^Yb atoms in a magnetic field. Using spin-polarized samples, we located several narrow magnetic Feshbach resonances between 580 and 700 G by detecting enhanced three-body recombination near these resonances. We showed that their locations can be quantitatively explained based on the most-accurate Born-Oppenheimer potential in the literature and our own *ab initio* calculation of a separation-dependent hyperfine coupling between the electron spin of ^6^Li and the nuclear spin of ^173^Yb.

A comparison of experimental and theoretical line profiles of the three-body recombination process at various temperatures has shown that recombination is controlled by *p*-wave scattering of the three-atom entrance channel. The observed temperature independence of the loss rate coefficient is unique to the fermionic quantum statistics of the collision partners and contrasts with the temperature-dependent behavior for *s*-wave and *d*-wave bosonic scattering [31]. The analysis has also shown that the maximum recombination rate coefficient is small compared to those found for Feshbach resonances in bosonic gases.

Our two-species experiments can reach temperatures below 1 *μ*K, where both species are quantum degenerate, by using a magnetic field gradient to counter their different gravitational potentials in order to maintain spatial overlap and thus maintain thermalizing elastic collisions between the two species throughout the cooling process [54,82]. At temperatures below quantum degeneracy we expect the resonance linewidth due to three-body recombination to become narrower than observed in this work, with a line profile that is modified by Fermi statistics. Improving magnetic field stability to be near 1 mG will be important in this regime.

Our observed MFRs give the highly mass-imbalanced ^173^Yb-^6^Li Fermi-Fermi mixture strong interactions for potential applications in few- and many-body physics, and are also expected to exist in other Yb-Li isotopologues involving ^173^Yb or ^171^Yb. Our results also provide a launch pad for the production of ultracold doublet ground-state molecules. This exciting prospect will require production of low entropy samples of ^6^Li and ^173^Yb in a three-dimensional optical lattice [83,84]. Under such conditions, combined with improved magnetic field stability, atom-molecule coupling will be sufficiently strong for efficient conversion of atoms into weakly bound molecules by a magnetic field sweep across one of our resonances [85].

## Figures and Tables

**TABLE I.** Observed ^173^Yb^6^Li Feshbach resonance locations and corresponding theoretical predictions and assignments based on *s*-wave coupled-channels calculations. The first two columns describe the quantum numbers of the scattering states and the projection of the total angular momentum, respectively. The third column gives the observed resonance locations defined as the *B* field of maximum atom loss for a sample at our lowest temperatures. Finally, the fourth and fifth columns give the resonance location and strength from the coupled-channels calculations. For all resonances the background scattering length is 30.4*a*_0_ and the magnetic moment difference between the molecular resonance and scattering channels is *δμ* = 1.99*μ_B_*. The error in the observed locations is the one-standard-deviation uncertainty from the quadrature sum of the statistical error in the Gaussian fit and the systematic error in the field calibration. The theoretical locations of the resonances have a 1.3 G one-standard-deviation uncertainty due to the uncertainty of the binding energy of the most weakly bound state. The differences between neighboring resonances are not affected by this uncertainty.

$|m_{s,\text{Li}}^{\text{at}},m_{i,\text{Li}}^{\text{at}}\rangle +|m_{i,\text{Yb}}^{\text{at}}\rangle$	*M*_tot_	$B_{\text{res}}^{\text{exp}}\;(G)$	$B_{\text{res}}^{\text{th}}\;(G)$	Δ (*μ*G)
∣−1/2, 1⟩ + ∣+5/2⟩	3	588.126(41)	587.803	28
∣−1/2, 0⟩ + ∣−3/2⟩	−2	640.161(40)	639.605	28
∣−1/2, 0⟩ + ∣−1/2⟩	−1	640.216(41)	639.670	44
∣−1/2, 0⟩ + ∣+1/2⟩	0	640.289(40)	639.736	50
∣−1/2, 0⟩ + ∣+3/2⟩	1	640.420(40)	639.802	44
∣−1/2, 0⟩ + ∣+5/2⟩	2	640.502(44)	639.870	28
∣−1/2, −1⟩ + ∣+5/2⟩	1	697.523(40)	696.545	28
